# CT-guided three-dimensional reconstruction with predictive modeling for personalized esophageal anastomosis in type C esophageal atresia

**DOI:** 10.3389/fsurg.2026.1871665

**Published:** 2026-09-14

**Authors:** Vassiliy Lozovoy, Zhenis Sakuov, Assylzhan Yerekeshov, Dastan Rustemov, Ruslan Bilal, Temirlan Zhangaskanov, Zariat Shatkenova

**Affiliations:** 1 Department of Pediatric Surgery, Astana Medical University, Astana, Kazakhstan; 2 Department of Neonatal Surgery, University Medical Center Corporate Fund, Astana, Kazakhstan; 3 Nazarbayev University School of Medicine, Astana, Kazakhstan; 4 Nazarbayev University School of Sciences and Humanities, Astana, Kazakhstan

**Keywords:** 3d (three-dimensional) image, computed tomography, esophageal atresia (EA), modified anastomosis, personalised surgery

## Abstract

**Background:**

Anastomotic stricture remains the most common and clinically significant complication following primary repair of type C esophageal atresia. Despite various technical modifications, postoperative narrowing at the anastomotic site continues to occur. We describe an approach incorporating preoperative three-dimensional CT reconstruction to enable individualized planning of the esophageal anastomosis.

**Aims and Objectives:**

To evaluate the feasibility, safety, and early clinical outcomes of a modified esophageal anastomosis guided by preoperative radiological and CT-based measurements in neonates with type C esophageal atresia.

**Materials and Methods:**

This single-center case series included five consecutive neonates who underwent primary repair between January 2022 and December 2023. Preoperative CT was performed to assess associated anomalies. Multiplanar and 3D reconstructions were used to measure the diameters of the proximal esophageal segment and the distal esophagus at the fistula level. Based on these parameters, a geometry-based calculation was applied preoperatively to determine the optimal length and configuration of the distal esophageal incision to achieve a wider anastomosis.

**Results:**

On postoperative day 7, contrast esophagography demonstrated a mean anastomotic diameter of 4.71 ± 0.43 mm. One patient developed an anastomotic leak requiring re-thoracotomy and repair. No cases of tracheoesophageal fistula recurrence were observed. During 12 months of follow-up, no patients developed clinically significant anastomotic stricture requiring gastrostomy or repeated dilatation.

**Conclusion:**

CT-based three-dimensional reconstruction combined with predictive modeling enables individualized incision planning and formation of a wider anastomosis. This approach may reduce postoperative strictures and support patient-specific management of type C esophageal atresia.

## Introduction

Esophageal atresia (EA) is a congenital condition defined by disrupted esophageal continuity, which may be accompanied by a tracheoesophageal fistula (TEF) (1). Esophageal atresia with a TEF (Gross type C) is the most common variant of this congenital malformation. Anastomotic stricture (AS) is the most common complication following repair of esophageal atresia type C (2) and frequently necessitates repeated postoperative bougienage procedures (3). The development of stricture is multifactorial, with surgical technique and anastomotic configuration being major contributing factors (4). Traditional circular end-to-end anastomoses are prone to contraction during healing, resulting in luminal narrowing. Although various technical modifications have been described, most do not allow precise adaptation to individual patient anatomy.

Diagnosis of esophageal atresia is based on the clinical presentation and confirmed by contrast radiography of the upper esophageal pouch. The routine use of computed tomography (CT) for preoperative evaluation of esophageal atresia is not recommended, but it is useful in the presence of associated pathologies, such as congenital heart defects, vascular anomalies, and other associated anomalies. In addition, 3D CT imaging can determine the level of the tracheoesophageal fistula and the distance between the atretic segments.

Using three-dimensional CT reconstruction, we determined the diameters of the proximal (D_1_) and distal (D_2_) esophageal pouches and used these anatomical parameters to define the planned anastomotic configuration (D_3_). Based on these patient-specific data, we developed a modified anastomotic technique guided by predictive mathematical modeling, aiming to achieve a wider lumen and potentially reduce the risk of postoperative stricture formation.

## Materials and methods

The study was approved by the local ethics committee, and written informed consent was obtained from the parents or legal guardians of all patients. This prospective, single-center observational study was conducted at the University Medical Center “Mother and Child Center” in Astana, Kazakhstan, between January 2022 and December 2023. Throughout this period, 25 newborns with esophageal atresia and a distal TEF were treated. Five patients underwent preoperative computed tomography because of associated anomalies. Specifically, a CT scan was performed in one patient for tracheal stenosis and in four patients as part of an evaluation for suspected congenital heart disease (Table 1).

**Table 1:** Summary of patient data and associated congenital anomalies.

№	Sex	Birth weight (g)	Associated anomaly
1.	M	3,170	Laryngeal stenosis
2.	M	3,450	Congenital heart defect + Anal atresia
3.	F	2,800	Congenital heart defect
4.	M	3,780	Congenital heart defect
5.	M	1,400	Congenital heart defect

### Preoperative imaging and measurements

Computed tomography was performed using a multislice CT scanner Aquilion Prime SP TSX-303B (Canon, Japan). Patients were positioned in the supine position to ensure stable and standardized imaging conditions. Images were reconstructed with a slice thickness of 0.6 mm and a reconstruction interval of 0.1 mm. Multiplanar reformatted images were generated interactively on a dedicated workstation (Vitrea Advanced Visualization) using the Lung Airway Analysis mode with the “airway lumen” (airway sinus) setting, allowing full 360-degree rotation. After CT imaging, three-dimensional reconstruction was carried out, allowing measurement of the diameters of the proximal (D_1_) and distal (D_2_) atretic esophageal segments (Figure 1). These two parameters (D_1_ and D_2_) were subsequently used for mathematical prediction of the optimal anastomotic configuration.

**Figure 1:**
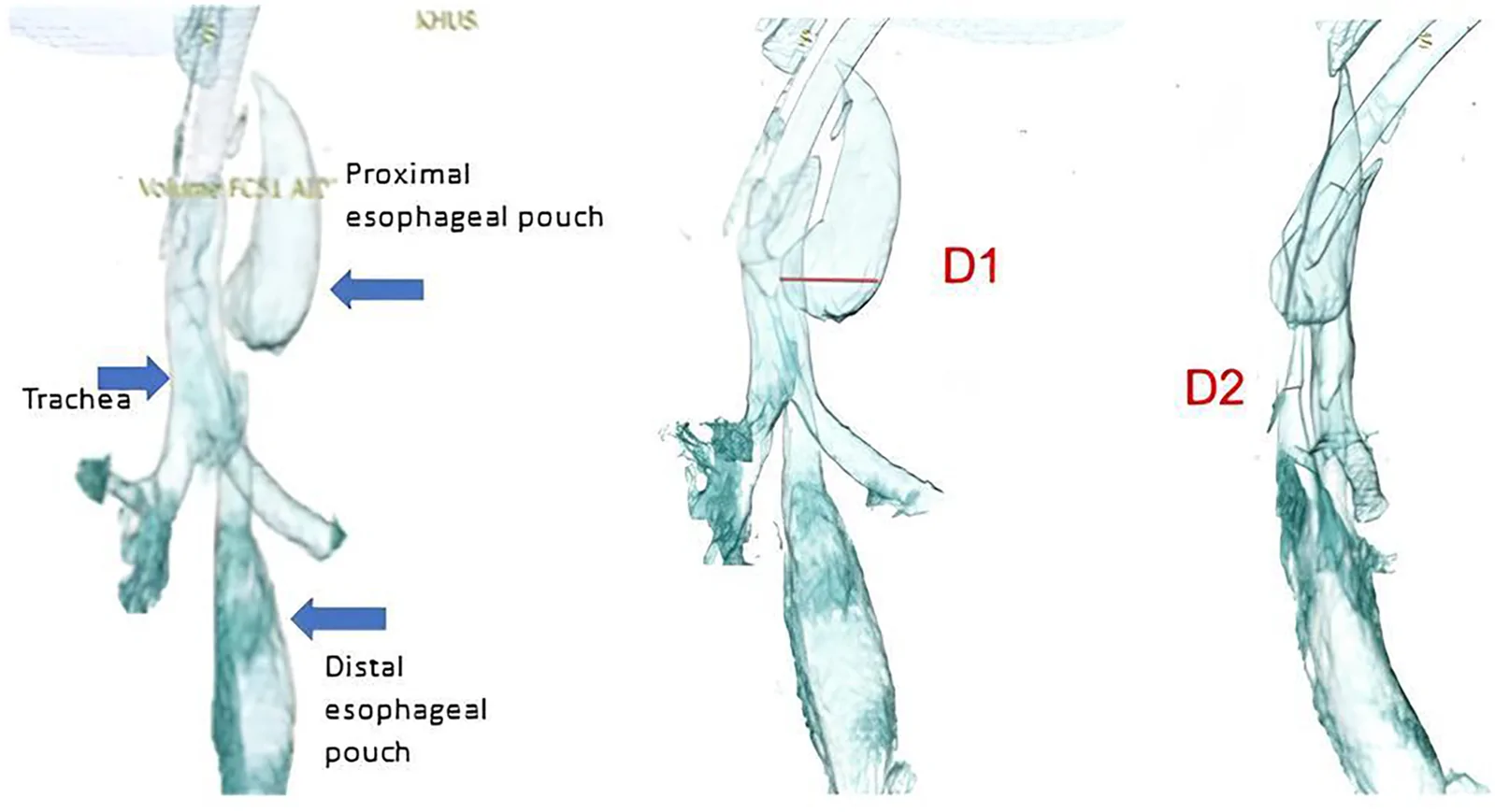
CT-based 3D model of the trachea and esophagus. D_1_, the diameter of the proximal esophageal segment; D_2_, the diameter of the distal esophageal segment.

CT examinations were performed using pediatric low-dose protocols in accordance with the ALARA (As Low As Reasonably Achievable) principle. Radiation dose parameters ranged from CTDIvol 1.16–14.0 mGy and DLP 15.92–45.0 mGy·cm. The variability in radiation dose reflected differences in patient body weight (1.4–3.78 kg) and the use of intravenous contrast-enhanced CT in selected patients when clinically indicated.

### Relevance of mathematical modeling

In the surgical treatment of esophageal atresia, the formation of an esophago-esophageal anastomosis between segments of different diameters remains one of the key technical challenges. Mismatch between the circumferences of the anastomotic edges may lead to local redistribution of tension and may contribute to anastomotic leakage and subsequent cicatricial stricture formation. In clinical practice, incisions on the distal esophageal segment are used to compensate for differences in diameter between the proximal and distal segments. However, the depth and length of these incisions are usually determined empirically, which reduces the reproducibility of the technique and increases dependence on the individual experience of the surgeon. Therefore, in the present study, a geometric model was developed to estimate the incision depth required to compensate for the difference in circumference between the proximal and distal esophageal segments based on objective anatomical measurements obtained from CT-based three-dimensional reconstruction.

### The model is based on the following assumptions

1. The cross-sectional shape of the esophageal segments at the level of the planned anastomosis approximates a circle.
2. The length of the anastomotic edge of each segment is approximated by its circumference.
3. The principal geometric mismatch between the anastomotic edges is related to the difference in segment diameters.
4. Distal esophageal incisions increase the available length of the distal anastomotic edge.
5. Within the simplified geometric model, an incision of depth L creates two additional incision margins, each with a length approximating L. Therefore, an incision of depth L is considered to provide approximately 2L of additional anastomotic edge length when the longitudinal axes of the two segments are aligned.
6. When the longitudinal axes of the connected segments form an angle *θ*, the effective component of the incision contributing to geometric compensation is assumed to be reduced in proportion to cos *θ*. Consequently, a longer incision is required to provide the same effective increase in anastomotic edge length as the angle increases.

The circumference of a tubular structure is determined by the standard geometric expression:

C=π×D

where C is the circumference of the segment, measured in millimeters (mm), and D is the diameter of the segment, also measured in millimeters (mm).

### Derivation of the calculation formula

Let:

D₁ = diameter of the larger proximal esophageal segment, mm;
D₂ = diameter of the smaller distal esophageal segment, mm;
C₁ = calculated circumference of the proximal esophageal segment, mm;
C₂ = calculated circumference of the distal esophageal segment, mm;
ΔC = difference between the proximal and distal segment circumferences, mm;
p = planned percentage of the geometric mismatch to be compensated by the incision, %;
c = coverage coefficient, dimensionless;
*θ* = acute angle between the longitudinal axes of the proximal and distal esophageal segments, degrees (°);
Treq = theoretical total incision depth required to achieve the planned geometric compensation, mm;
L₁ = depth of the first standardized incision, mm;
L₂ = calculated depth of the additional second incision, mm.

The circumferences of the proximal and distal esophageal segments are calculated as:

$$C_{1}\;=\pi \times D_{1}$$

and:

$$C_{2}\;=\pi \times D_{2}$$

The difference in circumference between the two segments is therefore:

$$\Delta C\;=C_{1}-C_{2}$$

or:

$$\Delta \;=\pi \times (D_{1}-D_{2})$$

### Coverage coefficient

The planned percentage of the geometric mismatch to be compensated by the incision is represented by p.

The coverage coefficient c is defined as:

c=p/100

Thus:

p = 100% corresponds to c = 1.0
p = 75% corresponds to c = 0.75
p = 50% corresponds to c = 0.50

A value of p = 100% therefore indicates that the planned increase in distal anastomotic edge length is intended to compensate for the entire calculated circumferential difference between the larger proximal segment and the smaller distal segment.

The target increase in distal anastomotic edge length is consequently:

ΔCtarget=c×ΔC

Substituting the expression for ΔC:

$$\Delta \mathrm{Ctarget}=c\times \pi \times (D_{1}-D_{2})$$

In all five patients included in the present series, complete geometric compensation was planned. Therefore:

p=100%

and:

c=1.0

### Angular correction

The longitudinal axes of the proximal and distal esophageal segments may not always be aligned. To account for this spatial relationship, *θ* is defined as the acute angle between the longitudinal axes of the two segments.

When *θ* = 0°, the segments are considered axially aligned.

Within the simplified geometric model, when an incision is oriented at an angle *θ* relative to the direction in which geometric compensation is required, the effective component of an incision of depth L is assumed to be:

Leffective=L×cosθ

Because each incision creates two margins, the effective increase in available anastomotic edge length is therefore:

2×L×cosθ

To achieve the target increase in edge length, the following relat

2×Treq×cosθ=ΔCtarget

Substituting the expression for ΔCtarget:

$$2\times \mathrm{Treq}\times \cos\theta =c\times \pi \times (D_{1}-D_{2})$$

Solving for Treq gives:

$$\text{Treq = }\left[\text{c}\times \pi \times \left(D_{1}-D_{2}\right)\right]/2\times \cos\theta ]$$

Thus, the angular correction factor is:

kθ=1/cosθ

and the same equation may be expressed as:

$$\text{Treq = }\left[\text{c}\times \pi \times \left(D_{1}-D_{2}\right)\right]/2\times K\theta$$

As *θ* increases, cos *θ* decreases and the angular correction factor increases. Consequently, the calculated incision depth increases.

When:

$$\theta =0^{\circ }$$

then:

$$\cos\;0^{\circ }=1$$

and:

kθ=1

Therefore, for axially aligned segments, the formula reduces to:

$$\text{Treq = }\text{c}\times \pi \times \left(D_{1}-D_{2}\right)/2$$

For complete geometric compensation, where c = 1:

$$\text{Treq = }\pi \times \left(D_{1}-D_{2}\right)/2$$

The angular correction is defined for an acute angle:

$$0^{\circ }\leq \theta <90^{\circ }$$

The formula should not be extrapolated to *θ* approaching 90°, because the correction factor 1/cos *θ* increases rapidly and becomes mathematically undefined at 90°. Such configurations would require separate geometric and clinical evaluation rather than direct application of the present model.

In all five patients included in the present series, the measured angle between the longitudinal axes was 0°. Therefore:

$$\theta =0^{\circ }$$

cosθ=1

and:

kθ=1

Consequently, the angular correction did not alter the calculations in any of the five patients. The angular term is included as a geometric extension of the model for possible non-zero angular configurations in future patients. Its clinical applicability has not been evaluated in the present case series and requires further mathematical and clinical validation.

### Final calculation of the required incision

The theoretical total incision depth required for the planned geometric compensation is calculated as:

$$\text{Treq = }\left[\text{c}\times \pi \times \left(D_{1}-D_{2}\right)\right]/\left[2\times \cos\theta \right]$$

where:

Treq = theoretical total required incision depth, mm;
D₁ = diameter of the larger proximal esophageal segment, mm;
D₂ = diameter of the smaller distal esophageal segment, mm;
c = coverage coefficient, calculated as p/100;
*θ* = acute angle between the longitudinal axes of the connected esophageal segments.

For the patients included in the present series:

c=1.0

and:

$$\theta =0^{\circ }$$

Therefore:

$$\text{Treq = }\pi \times \left(D_{1}-D_{2}\right)/2$$

### Relationship between the model-derived requirement and the standardised first incision

In the operative technique used in this series, the first distal esophageal incision (L₁) was standardised at 5 mm. The 5-mm first incision represented the operative practice used in these patients and was not itself derived from the mathematical model.

Thus:

$$L_{1}=5\mathrm{mm}$$

The mathematical calculation was used to determine whether an additional second incision was required after accounting for the standardized first incision.

The depth of the second incision was calculated as:

$$L{}_{2}=\max\left(0,\;\mathrm{Treq}-L{}_{1}\right)$$

Since:

$$L_{1}=5\mathrm{mm}$$

the equation becomes:

$$L{}_{2}=\max\left(0,\;\mathrm{Treq}-5\right)$$

Substituting the complete geometric equation:

$$L{}_{2}=\max\left\{0,\left[c\times \pi \times \left(D{}_{1}-D{}_{2}\right)\right]/\left[2\times \cos\;\theta \right]-5\right\}$$

If:

Treq>5mm

a second incision was required, and its calculated depth was:

$$L{}_{2}=\mathrm{Treq}-5$$

If:

Treq≤5m

the calculated depth of the second incision was:

$$L{}_{2}=0\mathrm{mm}$$

and no second incision was performed.

It is important to distinguish between the theoretical model-derived incision requirement and the total incision depth used under the operative protocol.

The total planned incision depth was:

$$\mathrm{Tplan}=L{}_{1}+L{}_{2}$$

or:

Tplan=max(5,Treq)

Therefore, when Treq ≥ 5 mm, the planned total incision corresponded to the theoretical model-derived requirement.

When Treq < 5 mm, the standardized 5-mm first incision exceeded the theoretical geometric requirement. In these cases, no additional incision was performed.

This occurred in two patients in the present series. Their model-derived requirements were 3.94 mm and 2.54 mm, respectively, whereas the standardized first incision remained 5 mm. Therefore, the standardized incision exceeded the theoretical calculated requirement by 1.06 mm and 2.46 mm in these patients.

Because the 5-mm first incision was based on operative practice rather than on the geometric model, the clinical significance of this difference cannot be determined from the present small case series and represents a limitation of the current technique and model.

### Example of calculation

To illustrate the application of the model, the following anatomical parameters from one patient were used:

$$D_{1}=8.23\mathrm{mm}$$

$$D_{2}=3.25\mathrm{mm}$$

$$L_{1}=5\mathrm{mm}$$

p=100%

$$\theta =0^{\circ }$$

First, the coverage coefficient is calculated:

c=p/100

c=100/100

c=1.0

The difference between the proximal and distal segment diameters is:

$$D_{1}-D_{2}=8.23-3.25$$

$$D_{1}-D_{2}=4.98\mathrm{mm}$$

The difference in circumference is:

$$\Delta C=\pi \times \left(D{}_{1}-D{}_{2}\right)$$

ΔC=π×4.98

ΔC≈15.65mm

The angular correction factor is:

kθ=1/cosθ

Because:

$$\theta =0^{\circ }$$

then:

$$\cos\;0^{\circ }=1$$

and:

kθ=1

The theoretical total required incision depth is therefore:

$$\mathrm{Treq}=\left[c\times \pi \times \left(D{}_{1}-D{}_{2}\right)\right]/\left[2\times \cos\theta \right]$$

Substituting the values:

$$\mathrm{Treq}=[1.0\times \pi \times (8.23-3.25)]/[2\times \cos\;0^{\circ }]$$

Treq=[π×4.98]/2

Treq≈7.82mm

The required depth of the second incision is:

$$L{}_{2}=\mathrm{Treq}-L{}_{1}$$

$$L{}_{2}=7.82-5$$

$$L{}_{2}=2.82\mathrm{mm}$$

Since L₂ > 0, a second incision was required. The operative plan therefore consisted of the standardized first incision of 5 mm and an additional calculated incision of approximately 2.8 mm.

### Surgical technique

The neonate was positioned in the left lateral decubitus position with the right arm elevated. Under general anesthesia, a right thoracotomy was performed through the fourth intercostal space. The surgical field was accessed via an extrapleural approach. The azygos vein (preserved) and the TEF were identified, after which the fistula was ligated and divided.

Following adequate mobilization of both the proximal and distal esophageal segments, the proximal pouch was opened using a transverse incision. The first distal esophageal incision (L_1_) was standardized at 5 mm in all patients and was planned preoperatively. The length of the second incision (L_2_) was then calculated preoperatively using the predictive formula based on the measured D_1_ and D_2_ values. In cases where the distal lumen was considered sufficiently enlarged after the initial 5-mm incision, no additional L_2_ incision was required. The calculated incision lengths were measured using a sterile millimeter ruler. Both L_1_ and L_2_ were marked on the distal esophageal segment according to the preoperative calculations before performing the incisions.

This approach allowed enlargement of the distal esophageal lumen, facilitating the creation of a wider anastomosis in more than one plane (Figure 2). The anastomosis was constructed using interrupted absorbable sutures (polydioxanone 5-0), placed to minimize circumferential tension. A nasogastric tube was passed across the anastomosis. The incision was closed in layers following the placement of an intercostal drainage tube in all cases.

**Figure 2:**
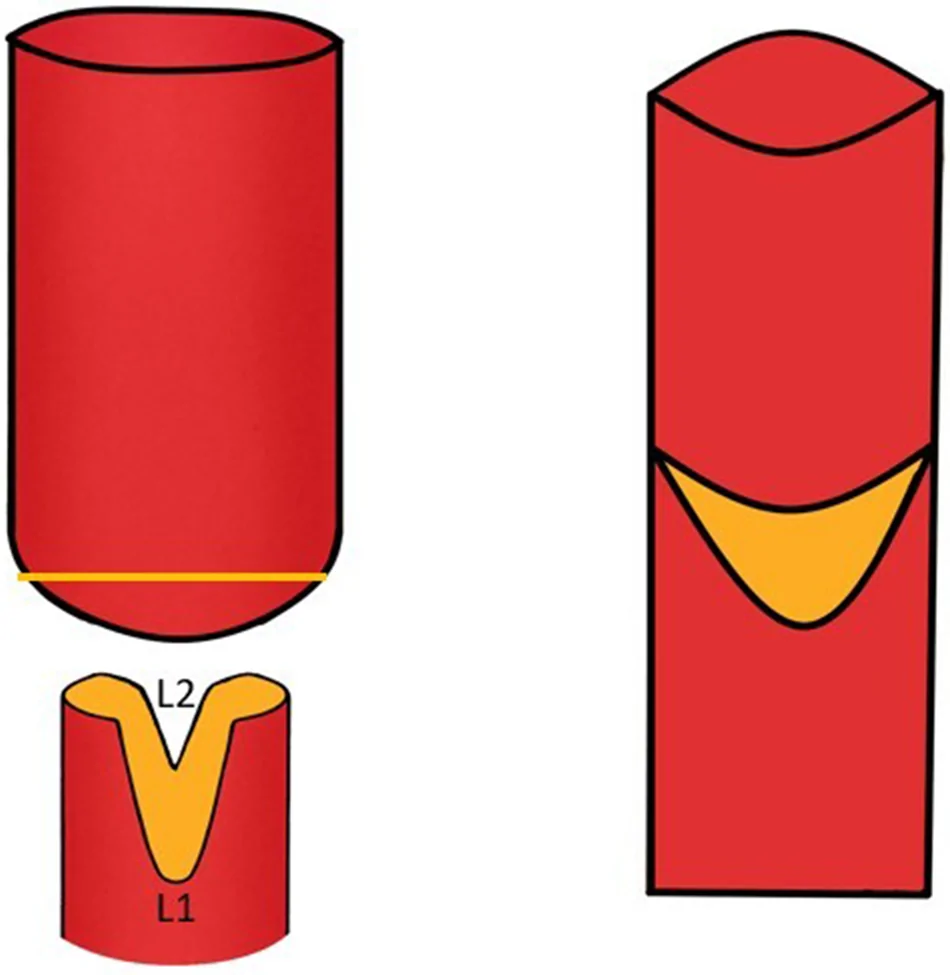
Schematic illustration of the incision markings and the final configuration of the anastomosis. L₁, depth of the first incision of the esophageal wall. L₂, depth of the second incision of the esophageal wall.

### Postoperative evaluation

All patients received standardized postoperative care, including nasogastric decompression, administration of broad-spectrum antibiotics, and parenteral nutrition until enteral feeding was established. All patients received postoperative mechanical ventilatory support during the early postoperative period according to our institutional protocol for EA repair. Postoperative anastomotic diameter (D_3_) was assessed on postoperative day 7 using contrast esophagography. Measurements were performed by the operating pediatric surgeon using digital imaging software. Each measurement was repeated three times, and the mean value was used for analysis (Figure 3). In the absence of anastomotic complications, the chest drain was subsequently removed. Anastomotic leakage was defined as extravasation of contrast material, whereas stricture and recurrence of tracheoesophageal fistula were diagnosed based on clinical symptoms, including dysphagia, regurgitation, and recurrent respiratory infections, and confirmed by radiological imaging.

**Figure 3:**
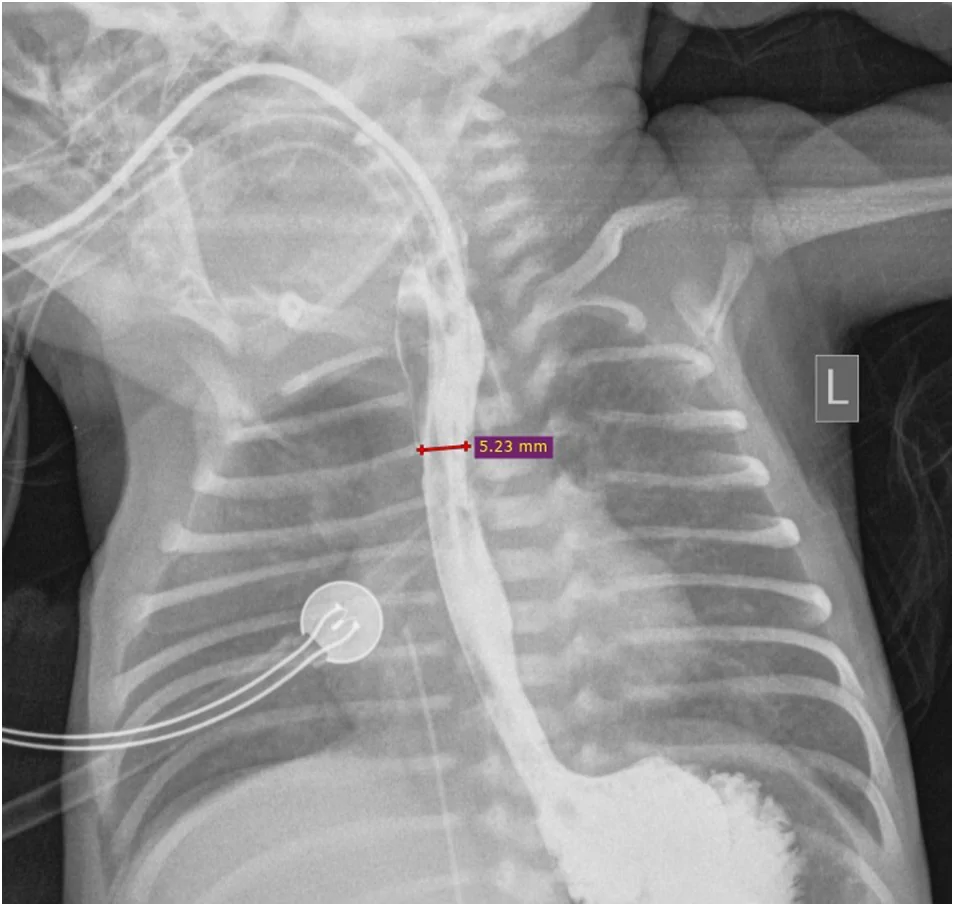
Measurement of the esophageal anastomotic diameter (D_3_).

### Follow-up

All patients were followed prospectively for at least 12 months after surgery. The primary outcome was the postoperative anastomotic diameter (D_3_). Secondary outcomes included anastomotic leakage, recurrent TEF, anastomotic stricture requiring esophageal dilatation or gastrostomy, and clinically evident chest wall deformity.

## Results

Five neonates with esophageal atresia type C were included in this case series. All five patients had associated congenital anomalies; however, none had a confirmed syndromic diagnosis (Table 1). Prenatal diagnosis of EA was established in two patients. In two additional patients, the diagnosis was not suspected antenatally. One patient had prenatal polyhydramnios without a definitive diagnosis of EA before birth. All patients underwent primary repair during the neonatal period using the modified CT-guided anastomotic technique. The results are presented in Table 2.

**Table 2:** Comparison of esophageal segments diameters, calculated incision parameters, and clinical outcomes.

No.	D₁ (mm)	D₂ (mm)	*p* (%)	c	*θ* (°)	Treq (mm)	L₁ (mm)	Calculated L₂ (mm)	Second incision performed	Planned total incision (mm)	D₃ (mm)	Δ (%)	Leakage
1	9.37	3.81	100	1	0	8.73	5.00	3.73	Yes	8.73	5.01	31.5	Yes
2	10.08	3.32	100	1	0	10.62	5.00	5.62	Yes	10.62	4.42	33.1	No
3	6.01	3.50	100	1	0	3.94	5.00	0.00	No	5.00	4.16	18.9	No
4	8.23	3.25	100	1	0	7.82	5.00	2.82	Yes	7.82	4.71	44.9	No
5	6.05	4.43	100	1	0	2.54	5.00	0.00	No	5.00	5.23	18.1	No
Mean	7.95	3.66	100	1	0	6.73	5.00	2.43	3/5	7.43	4.71	29.3	1/5

D₁ — diameter of the proximal esophageal segment.

D₂ — diameter of the distal esophageal segment before anastomosis.

p — planned percentage of the calculated circumferential mismatch to be compensated.

c — coverage coefficient calculated as:

c=p/100.

In all five patients:

p=100%.

and:.

c=1.00.

θ — acute angle between the longitudinal axes of the proximal and distal esophageal segments. The measured angle was 0° in all patients in the present series.

Treq — theoretical total incision depth required to achieve the planned geometric compensation, calculated as: $\mathrm{Treq}=\left[c\times \pi \times \left(D{}_{1}-\;D{}_{2}\right)\right]/\left[2\times \cos\;\theta \right].$

L₁ — depth of the first standardized distal esophageal incision. The 5-mm value was based on the operative practice used in this series rather than on the mathematical model.

L₂ — calculated depth of the additional second incision, determined as: $L{}_{2}=\max\left(0,\;\mathrm{Treq}\;-L{}_{1}\right).$

Second incision performed — indicates whether an additional distal incision was performed after the standardized first incision.

Planned total incision — sum of the standardized first incision and the calculated additional incision: $\mathrm{Tplan}=L{}_{1}+L{}_{2}$

D₃ — postoperative anastomotic diameter measured by contrast esophagography on postoperative day 7.

Δ (%) — percentage increase in anastomotic diameter relative to the initial distal esophageal diameter D₂, calculated as:. $\Delta \left(\%\right)=[\left(D{}_{3}-D{}_{2}\right)/D{}_{2}]\times 100.$

Because θ = 0° in all five patients, the angular correction factor was equal to 1 and did not affect the calculations in the present series. The angular component of the model represents a theoretical geometric extension intended for future patients with non-zero angular configurations and requires further mathematical and clinical validation.

In patients 3 and 5, Treq was less than the standardized 5-mm first incision. Consequently, no second incision was performed, although the total incision used under the operative protocol exceeded the theoretical geometric requirement in these two patients.

Table 2 summarizes the esophageal diameters and clinical outcomes in the study group. The initial distal esophageal incision (L_1_) was standardized at 5 mm in all patients. In three patients, the predictive model indicated that an additional incision (L_2_) was required and was subsequently performed. In the remaining two patients, the calculated L_2_ value was negative, indicating that the enlargement achieved by the standardized 5-mm L_1_ incision alone exceeded the minimum calculated requirement for distal lumen enlargement. Because a negative incision length has no surgical meaning, no second incision was performed in these cases. The mean proximal esophageal diameter (D_1_) was 7.9 mm, whereas the distal esophageal diameter (D_2_) remained smaller, with a mean value of 3.66 mm. The mean anastomotic diameter (D_3_) measured 4.70 ± 0.43 mm (*n* = 5), with a median of 4.71 mm. The values ranged from 4.16 to 5.23 mm, and the interquartile range was 4.29–5.12 mm. The percentage increase in anastomotic diameter relative to the distal esophageal diameter (Δ) averaged 29.3%, ranging from 18.1% to 44.9%. One patient developed an anastomotic leak that, despite conservative treatment, required re-thoracotomy and repair of the defect. This patient remained free of dysphagia during follow-up. None of the patients developed dysphagia or other symptoms suggestive of anastomotic stricture. Consequently, no patient required esophageal dilatation or gastrostomy placement during the 12-month follow-up period. No cases of recurrent tracheoesophageal fistula or clinically evident chest wall deformity were observed.

## Discussion

Anastomotic stricture (AS) is the most common postoperative complication following type C esophageal atresia repair and may require repeated dilatation procedures (5). Its development is multifactorial: pre-operative factors such as gap length and prematurity, intraoperative factors including anastomotic tension and suture technique, and postoperative issues like anastomotic leak and gastroesophageal reflux all contribute (4). Importantly, the anastomotic configuration itself has a major impact. Traditional circular end-to-end anastomosis tends to contract substantially during the healing process, reducing the luminal diameter and predisposing to stricture. Consequently, various modifications of the anastomotic technique have been explored to minimize this complication (6).

P. Catalano and colleagues (2011) presented a technique for performing an oblique esophageal anastomosis to prevent the development of AS following surgical repair of EA. A retrospective review of 16 patients (14 neonates with type III and 2 with type IV esophageal atresia according to the Ladd-Gross classification) demonstrated that this method, which creates a wider suture line not confined to a single plane, effectively minimizes the risk of stricture formation. The study concluded that the oblique anastomosis technique is a safe and effective alternative to the conventional end-to-end esophageal anastomosis (4).

In the study by Mohamed Oulad Saiad, a novel surgical technique was evaluated, designed to minimize the risk of AS following esophageal atresia repair. A retrospective analysis of 49 patients was conducted, comparing outcomes between those who underwent a wave-shaped anastomosis and those who received a traditional end-to-end anastomosis. The results showed that none of the patients in the first group developed strictures, whereas eight patients in the second group did, representing a statistically significant difference (*p* = 0.046). The technique is characterized by a wide, multi-planar anastomosis that creates a large low-tension zone, promoting better healing and reducing the likelihood of stricture formation. The authors suggested that further studies are required to confirm these findings (7).

Another article introduced a new technique for esophageal anastomosis aimed at minimizing the risk of AS formation in patients with EA. The method involves creating a wide anastomosis with a suture line not confined to a single plane, which allows for uniform tension distribution and reduces the likelihood of narrowing during the healing process. Over a 10-year period, this method was applied in 11 cases, with only one patient developing an AS, which was successfully managed with a single dilatation (8).

In a different study, a novel surgical technique for primary repair of EA was presented, intended to prevent AS formation. The authors, Mehmet Melek and Ufuk Cobanoglu, described a method involving a plus-shaped (+) incision at the proximal end of the atretic esophagus, which allows for an expanded anastomotic area. This technique was applied to 11 patients with EA and tracheoesophageal fistula between 2005 and 2009. The results demonstrated that none of the patients required dilatation for AS and none experienced symptomatic dysphagia during a four-year follow-up period. The authors concluded that this method minimizes stricture formation and provides an effective approach for esophageal reconstruction in infants (9).

Although routine CT imaging is not generally recommended for diagnosing esophageal atresia in newborns (10), it can help identify the level of the distal tracheoesophageal fistula and reduce intraoperative difficulties during its dissection, as well as reveal additional anomalies such as a right-sided aortic arch, vertebral anomalies, pulmonary abnormalities, and other congenital anomalies (11). To the best of our knowledge, no previous study has reported on tailoring the type of anastomosis in esophageal atresia to an individual patient based on CT 3D modeling, even though current technological advancements make this approach feasible.

It should be emphasized that several previously described modifications of esophageal anastomosis also incorporate distal esophageal incisions to enlarge the lumen. However, in most of these techniques, the depth and length of the incisions are determined intraoperatively and rely heavily on the surgeon's subjective assessment and experience. In contrast, the key advantage of the proposed approach is the ability to define incision parameters preoperatively through mathematical modeling based on three-dimensional CT reconstruction. This strategy provides a more objective method of surgical planning and supports the creation of a tailored, patient-specific anastomosis that accounts for individual anatomical variability. However, a notable limitation of CT-based planning is the associated radiation exposure, which is of particular concern in the neonatal population and should be carefully considered when selecting patients for this approach.

In our series, a CT-based 3D reconstruction allowed successful esophageal anastomosis in all five patients. The present data demonstrate that the anastomotic diameter (D_3_) consistently exceeded the distal esophageal diameter (D_2_), with a mean increase of 29.3%. This finding suggests that the applied surgical approach allows for effective enlargement of the distal segment and the formation of a relatively wide anastomosis, which is hypothesized to contribute to a lower risk of postoperative stricture formation. During the 12-month follow-up period, none of the patients developed dysphagia, and none required esophageal dilatation.

One patient developed an anastomotic leak requiring reoperation. Although this patient had significant associated pathology, including congenital laryngeal stenosis and prolonged respiratory compromise, the potential contribution of the modified anastomotic technique cannot be completely excluded. Enlargement of the distal esophagus through additional incisions may compromise local vascular supply or increase tension at the anastomotic margin, thereby increasing the risk of leakage. Furthermore, it is possible that a conventional anastomosis without distal esophageal enlargement would have resulted in a different balance between leakage risk and subsequent stricture formation. Given the small sample size, the observed leak should be interpreted cautiously, and larger comparative studies are required to determine the safety profile of this technique.

Recent advances in artificial intelligence, computer vision, and predictive surgical analytics are facilitating personalized operative planning and intraoperative decision-making. While the present study does not utilize machine learning algorithms, it applies a patient-specific quantitative planning strategy based on three-dimensional anatomical reconstruction. Such approaches may represent an early step toward future AI-assisted surgical planning systems in neonatal surgery. Furthermore, advances in computer vision and thoracoscopic image analysis may eventually enable real-time intraoperative measurements, potentially reducing dependence on preoperative CT imaging and further supporting personalized esophageal reconstruction strategies (12, 13).

The present study was designed as a case series and did not include a concurrent control group. Therefore, the absence of anastomotic strictures during follow-up should be interpreted cautiously. Published studies have reported anastomotic stricture rates ranging from 30% to 50% following conventional repair of type C esophageal atresia. While no strictures were observed in our cohort, the small sample size and uncontrolled study design do not allow conclusions regarding superiority of the proposed technique. Larger comparative studies are needed to validate these preliminary findings.

### Limitations

The proposed mathematical model has several limitations:

1. It does not account for tissue heterogeneity of the esophageal wall.
2. It does not include biomechanical parameters (elastic modulus, stress distribution).
3. It is mainly applicable to primary esophago-esophageal anastomosis.

Several limitations should be acknowledged regarding D_3_ assessment. Preoperative diameters (D_1_ and D_2_) were obtained using CT-based three-dimensional reconstruction, whereas postoperative diameter (D_3_) was measured using contrast esophagography. These imaging modalities differ in spatial resolution and measurement characteristics. Furthermore, esophagographic measurements may be influenced by the degree of luminal distension, contrast volume, patient positioning, and respiratory motion at the time of image acquisition. Although each D_3_ measurement was performed three times and averaged, no formal inter-observer or intra-observer reliability analysis was conducted.

The occurrence of one anastomotic leak in this small cohort limits conclusions regarding the safety of the technique and does not allow exclusion of a possible association between the additional distal esophageal incisions and postoperative leakage.

## Conclusion

Integration of CT-based three-dimensional reconstruction with predictive mathematical modeling enables individualized planning of the anastomotic incision and facilitates construction of a wider anastomosis. This approach may reduce the incidence of postoperative strictures, potentially decrease the need for repeated esophageal bougienage, and could provide a patient-specific strategy for the surgical correction of type C esophageal atresia.

## Data Availability

The raw data supporting the conclusions of this article will be made available by the authors, without undue reservation.
